# Dietary Strawberries Improve Cardiometabolic Risks in Adults with Obesity and Elevated Serum LDL Cholesterol in a Randomized Controlled Crossover Trial

**DOI:** 10.3390/nu13051421

**Published:** 2021-04-23

**Authors:** Arpita Basu, Kenneth Izuora, Nancy M. Betts, Jefferson W. Kinney, Arnold M. Salazar, Jeffrey L. Ebersole, R. Hal Scofield

**Affiliations:** 1 Department of Kinesiology and Nutrition Sciences, School of Integrated Health Sciences, University of Nevada, Las Vegas, NV 89154, USA; 2 Section of Endocrinology, School of Medicine, University of Nevada, Las Vegas, NV 89154, USA; kenneth.izuora@unlv.edu; 3 Department of Nutritional Sciences, Oklahoma State University, Stillwater, OK 74078, USA; nancy.betts@okstate.edu; 4 Department of Brain Health, School of Integrated Health Sciences, University of Nevada, Las Vegas, NV 89154, USA; Jefferson.Kinney@unlv.edu (J.W.K.); arnold.salazar@unlv.edu (A.M.S.); 5 School of Dental Medicine, University of Nevada, Las Vegas, NV 89154, USA; jeffrey.ebersole@unlv.edu; 6 Section of Endocrinology and Diabetes, University of Oklahoma Health Sciences Center, Oklahoma City, OK 73104, USA; Hal-Scofield@omrf.org; 7 Arthritis and Clinical Immunology, Oklahoma Medical Research Foundation, Oklahoma City, OK 73104, USA

**Keywords:** strawberries, obesity, insulin resistance, LDL cholesterol, small LDL particles, plasminogen activator inhibitor-1

## Abstract

**Background and aims:** Dietary berries, such as strawberries, are rich in bioactive compounds and have been shown to lower cardiometabolic risk. We examined the effects of two dietary achievable doses of strawberries on glycemic control and lipid profiles in obese adults with elevated serum LDL cholesterol (LDL-C). **Methods:** In this 14-week randomized controlled crossover study, participants were assigned to one of the three arms for four weeks separated by a one-week washout period: control powder, one serving (low dose: 13 g strawberry powder/day), or two-and-a -half servings (high dose: 32 g strawberry powder/day). Participants were instructed to follow their usual diet and lifestyle while refraining from consuming other berries and related products throughout the study interval. Blood samples, anthropometric measures, blood pressure, and dietary and physical activity data were collected at baseline and at the end of each four-week phase of intervention. **Results:** In total, 33 participants completed all three phases of the trial [(mean ± SD): Age: 53 ± 13 y; BMI: 33 ± 3.0 kg/m^2^). Findings revealed significant reductions in fasting insulin (*p* = 0.0002) and homeostatic model of assessment of insulin resistance (*p* = 0.0003) following the high dose strawberry phase when compared to the low dose strawberry and control phases. Glucose and conventional lipid profiles did not differ among the phases. Nuclear magnetic resonance-determined particle concentrations of total VLDL and chylomicrons, small VLDL, and total and small LDL were significantly decreased after the high dose strawberry phase, compared to control and low dose phases (all *p* < 0.0001). Among the biomarkers of inflammation and adipokines measured, only serum PAI-1 showed a decrease after the high dose strawberry phase (*p* = 0.002). **Conclusions:** These data suggest that consuming strawberries at two-and-a-half servings for four weeks significantly improves insulin resistance, lipid particle profiles, and serum PAI-1 in obese adults with elevated serum LDL-C.

## 1. Introduction

Historically, fruits and vegetables, as plant-based food groups, have been promoted for various health benefits, especially in the context of reducing risks of adiposity, type 2 diabetes, cancer, and cardiovascular disease (CVD) [1,2]. Several components in fruits and vegetables are identified for their physiological effects in reducing risks of chronic diseases among which the phytochemicals deserve special attention [3,4]. The benefits of commonly consumed whole berry fruits, especially blueberries and strawberries, have been emphasized for their distinct effects in improving metabolic syndrome, a constellation of diabetes and CVD risks [5]. These improvements are ascribed mainly via mediating weight loss and reducing blood pressure at recommended levels of intakes [6]. Most of the health benefits of berry fruits, especially blueberries, cranberries, and strawberries, have been explained by their high polyphenol and fiber content, and concomitant low caloric contributions to the diet [7,8]. Despite this scientific evidence on why whole fruits and vegetables must be essential food groups in the daily diet, US adults have continued low intake of fruits and vegetables associated with increased risk of type 2 diabetes and CVD [9]. To address these dietary deficiencies and to overcome the behavioral challenge of changing one’s entire habitual diet to a healthy diet, whole berries have been supplemented as functional foods in several clinical trials yielding favorable compliance and improvement in cardiometabolic risks.

Among several CVD risks widely observed in the global population, metabolic syndrome [10] with elevated blood lipids such as total and LDL cholesterol [11,12] remains prominent. Dietary berries have been demonstrated to reduce these risks in clinical trials [13,14]. However, the reported findings are inconsistent, and experimental heterogeneity among doses and forms of different berries, study duration, outcomes reported, and participant characteristics make concrete recommendations challenging. To address this issue, our group has reported a series of clinical studies, mainly to assess the optimal dose and effects of whole strawberries on cardiometabolic risk in adults with features of the metabolic syndrome [15,16,17]. In our previously reported studies, strawberry supplementation was shown to decrease total and LDL cholesterol in overall healthy adults with abdominal obesity and dyslipidemia [15,16]. Most of these lipid-lowering effects were observed in the group consuming a large dose of freeze-dried powder equivalent to a pound of fresh strawberries daily. Thus, we conducted the current trial using dietary achievable doses of strawberries, equivalent to one serving, and two-and-a-half servings of strawberries daily using a randomized controlled crossover design in obese adults and LDL-cholesterol above optimal levels. We hypothesized that dietary strawberry supplementation, administered as freeze-dried strawberry powder, will improve cardiometabolic risk in these adults when compared to the control group over a 14-week duration.

## 2. Materials and Methods

This is a multicenter, randomized, double-blind controlled, crossover trial conducted at the Oklahoma Clinical and Translational Sciences Institute (OCTSI) at the University of Oklahoma Health Sciences Center (OUHSC), Department of Nutritional Sciences at the Oklahoma State University (OSU), and the Section of Endocrinology at the University of Nevada at Las Vegas (UNLV) School of Medicine. The study was approved by the OUHSC, OSU, and UNLV Institutional Review Boards (IRB number 1119274) and was registered at Clinicaltrials.gov (Identifier: NCT03441620). All participants provided written informed consent.

### 2.1. Study Criteria and Protocol

Participants were recruited using the campus system of email communication to all faculty, staff, and students at each of the three sites, as well as by flyers posted at buildings, such as the student wellness centers and the academic advising centers on each campus. Adult men and women with one or more features of metabolic syndrome [5], abdominal adiposity (waist circumference: men >40 inches; women >35 inches), body mass index (BMI) in the obese range (≥30 kg/m^2^), and elevated serum LDL cholesterol (LDL-C) > 116 mg/dL were enrolled in the study. Exclusion criteria were current use of medications that may influence glucose and lipid metabolism (metformin, statins, glucocorticoids, immunosuppressants, antipsychotics), unwillingness or inability to provide written informed consent, or significant underlying medical disorder assessed by the study physician (e.g., anemia, renal disorders, diabetes), and smokers. Participants were also excluded if they were allergic to berries, were unwilling to make dietary changes, or were vegetarian or consuming any other special diet not consumed habitually. Using a crossover design, participants completed three, four-week interventions in a randomized counterbalanced order using a Latin Square Design. During each four-week period, participants consumed beverages reconstituted with water from control powder, or freeze-dried strawberry powders equivalent to one serving, or two-and-a-half servings of strawberries each day. There was a one-week washout phase between each phase. Participants were instructed to follow their usual diet and lifestyle habits throughout the 14-week study. Health and medical history, anthropometric measurements, blood pressure recordings, and blood draws were obtained at baseline and at the end of each four-week intervention phase. In addition to these visits, participants made biweekly short visits to the clinic to meet the registered dietitian (RD) and nurse practitioner, receive supplies of the test agents, and submit 24 h diet recalls.

### 2.2. Intervention and Control Powders

The composition of the control and freeze-dried strawberry powders, provided by the California Strawberry Commission (Watsonville, CA, USA) is shown in Table 1. These powders were provided to the participants in vacuum-sealed packets to be reconstituted with plain drinking water for consumption. The total volume of powder provided was similar for all three groups (~32 g/day) and was consumed in two separate doses at least six to eight hours apart. The participants were also asked to consume the reconstituted beverage by itself and not with a meal or other snacks to prevent the confounding effects of other dietary factors. Participants were provided with uniform instructions to consume the test beverage at least two hours after the main meal or snack, and preferably as a midmorning and early evening snack during the day. The control powder was formulated to match the sensory properties of the strawberry powder, as well as its caloric value and macronutrient composition. The nutrient and phytochemical composition of the strawberry and control powders was determined at the Robert M. Kerr Food and Agricultural Products Center at Oklahoma State University (Stillwater, OK, USA), and at the Brunswick Laboratories (Southborough, MA, USA), respectively.

### 2.3. Habitual Dietary Intake and Physical Activity Assessment

All study participants were asked to maintain a 24 h food recall each week, which they submitted to the RD during their biweekly visits and discussed any changes made in their habitual diet. All participants were otherwise expected to maintain their usual diet and level of physical activity throughout the study. Participants were also asked to refrain from consuming additional strawberries other than the freeze-dried powders provided in the study. Dietary analyses were conducted by the study RD or a trained dietetic assistant using the ESHA’s Food Processor^®^ Nutrition Analysis software for energy, nutrients, and food group intake for each participant. Self-reported physical activity participation was quantified by the International Physical Activity Questionnaire Short Form (IPAQ–SF), which assesses physical activity levels during the last seven days, and participant responses (yes/no) were recorded for meeting exercise recommendations (≥150 min of moderate and/or ≥90 min vigorous exercise/week) [18].

### 2.4. Compliance

Compliance was assessed based on the return of unused packets of the test powders at the end of each four-week phase of the study. Each participant also received weekly phone calls from the study team to ensure the timely consumption of control or strawberry beverages and to discuss general dietary concerns with the participants. Participants were also reminded to maintain their weekly 24 h food recalls. Serum ellagic acid was measured in the intervention groups as a marker of strawberry consumption using published methods [19]. The incidence and persistence of any side effects in the intervention groups, i.e., gastrointestinal symptoms and headaches were recorded.

### 2.5. Anthropometric Measures and Blood Pressure

Participant body weight, height, waist circumference, systolic and diastolic blood pressure were measured at baseline and at the end of each four-week phase of the trial. Body weight was measured using a digital scale in light clothing and no shoes. Systolic and diastolic blood pressure was measured in mm Hg using Spot Vital Signs Device (Welch Allyn, Skaneateles Falls, NY, USA). At each visit, participants were asked to lie down and relax for approximately 8–10 min, following which three blood pressure measurements were recorded at intervals of 5–8 min and mean values were recorded.

### 2.6. Biochemical Analyses

At each visit (baseline and end of each four-week phase) freshly drawn blood samples were sent to the University of Oklahoma Medical Center laboratory (Oklahoma City, OK, USA) or Quest Diagnostics (Las Vegas, NV, USA) for analyses of serum glucose and conventional lipid profiles, insulin, liver, and kidney function tests (Abbott Architect Instruments) with commercial kits, according to manufacturer’s protocols. C-reactive protein was assayed by ultrasensitive nephelometry (Dade Behring). Serum glycated hemoglobin was analyzed with the use of a DCA 2000+ Analyzer (Bayer). Insulin resistance was evaluated by HOMA-IR and was calculated as follows: [fasting insulin (mU/L) × fasting glucose (mmol/L)]/22.5 [20]. Nuclear magnetic resonance-determined lipoprotein subclass profile (NMR-LSP) was performed in first-thaw plasma specimens using a 400-MHz proton NMR analyzer at LipoScience Inc. (Morrisville, NC), as described previously [21]. In addition, sera were stored at −80 °C for the subsequent analyses of diabetes-related hormones and adipokines. Serum adiponectin was measured using a quantitative sandwich enzyme immunoassay technique (R&D Systems, Minneapolis, MN, USA) with an average intra-assay CV of 4.8%.

### 2.7. Human Diabetes Panel Assays

Diabetes-related adipokines and hormones were determined in available first-thaw serum samples at baseline and at the end of each four-week intervention phase. These were measured using Bio-Plex Pro™ Human Diabetes Panel magnetic bead assays (Bio-Rad Laboratories, Hercules, CA, USA), according to the manufacturer’s instructions. Frozen serum samples were thawed on ice and 1:3 dilutions were prepared with the sample diluent provided in the kit. Diluted samples were processed using the manufacturer’s protocol with the following modifications: (i) beads were incubated with the detection antibodies for 45 min; and (ii) beads were incubated with streptavidin–phycoerythrin antibody for 20 min. The variables measured and their corresponding intra-assay CVs were C-peptide ~4%, ghrelin ~4%, gastric inhibitory polypeptide (GIP) ~3%, glucagon-like peptide-1 (GLP-1) ~4%, glucagon ~5%, leptin ~3%, plasminogen activator inhibitor-1 (PAI-1) ~2.5%, resistin ~5%, and visfatin ~4%.

### 2.8. Statistical Analyses

All data were examined for normal distribution using Shapiro–Wilk test and Skewness/Kurtosis. No outliers were detected. Continuous variables were expressed as means ± SD and discrete variables as counts and percentages. We employed a mixed model ANOVA to examine the main effects of treatment, time, and interaction to examine differences in outcomes at the end of each four-week phase of intervention. Baseline values were included as covariates for each outcome variable. Outcomes were modeled as repeated measures, with subject as a random effect and with unstructured variance for treatment/time. When time and treatment-by-time interactions were nonsignificant, they were removed from the model. When time effects were significant, they were retained in the final model of treatment effects. The sequence of intervention was included in all models to test for carryover effects, and none were detected. Our previous strawberry study in adults with the metabolic syndrome [15,16], indicated a difference in serum LDL cholesterol of 12 ± 8 mg/dL (mean ± SD) and of small LDL particle concentrations of 108 ± 52 nmol/L would be expected using a sample size of 16/group within the four weeks of strawberry treatment to achieve 80% power at 0.05 α level. Considering a 20–30% attrition, we aimed to recruit and retain at least 21 participants in the crossover study. All *p*-values were two-tailed, and main effects and interaction effects were considered if <0.05. Analyses were performed using SAS (version 9.4; SAS Institute Inc., Cary, NC, USA).

## 3. Results

### 3.1. Baseline Characteristics and Compliance

**Figure 1:**
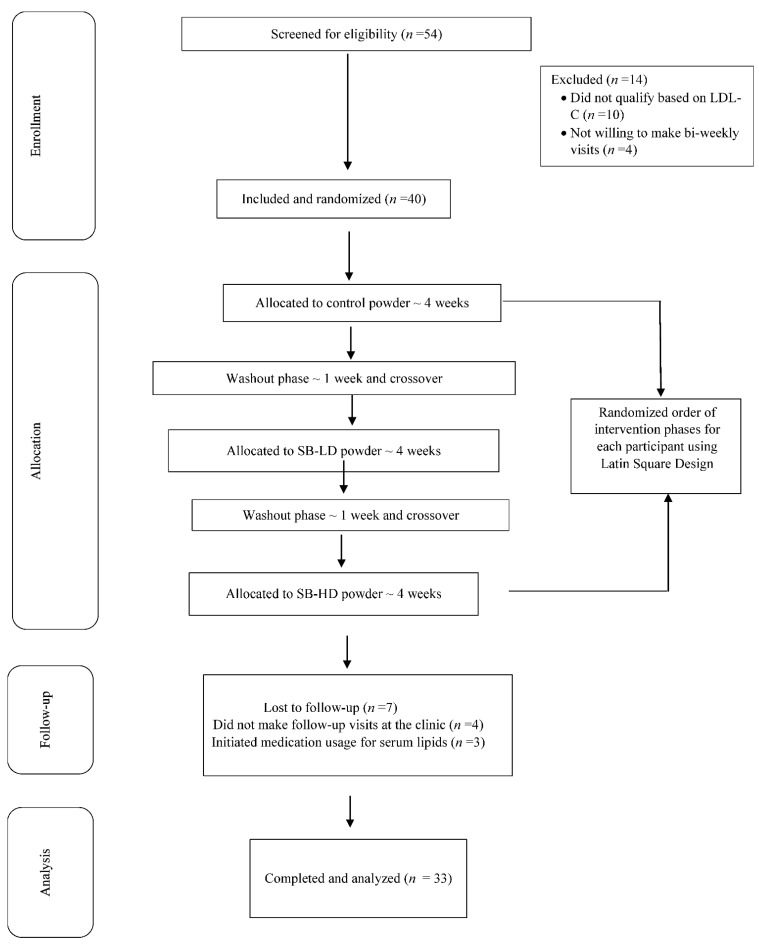
Study design. LDL-C: LDL cholesterol; SB–LD: low-dose strawberry phase; SB–HD: high-dose strawberry phase.

The study enrollment is shown in Figure 1. Table 2 presents the baseline characteristics of the 33 participants who completed all three phases of the randomized crossover study. Based on the completion of self-reported daily consumption logs and return of unused powder, protocol compliance was 96%. In addition, ellagic acid was measured in serum samples and revealed the following levels (means ± SD): baseline: not detectable; control: not detectable; low-dose strawberry: 15 ± 8 nmol/L; high-dose strawberry: 27 ± 13 nmol/L.

### 3.2. Anthropometrics, Blood Pressure, Glucose, Insulin, and Conventional Lipids

Among the biomarkers of glycemic control, a significant main effect of treatment was observed for serum insulin and insulin resistance (HOMA-IR), which revealed significantly lower values after the high dose strawberry phase, compared to values in the baseline, control, and low dose strawberry phases in post hoc analyses (*p* = 0.0002 and *p* = 0.0003, respectively; Table 3). No treatment effects were noted for serum glucose and HbA1c. In the conventional lipid profile, a borderline significant effect of treatment was noted for serum LDL cholesterol, which revealed lower values after the high-dose strawberry phase, compared to values in the baseline, control, and low-dose strawberry phases in post hoc analyses (*p* = 0.05; Table 3). No treatment effects were noted with serum total and HDL cholesterol and triglycerides. Similarly, no main effects of treatment were noted for body weight, BMI, and waist circumference, as well as systolic and diastolic blood pressure related to the strawberry intervention. Overall, treatment-by-time interactions were not significant for any of the outcomes except for serum insulin (*p* = 0.005), indicating that the effect of the treatment on insulin depended on the order in which the treatments were administered.

### 3.3. NMR-Derived Lipid Particle Profiles

Among the NMR-derived lipid particle concentrations and size, several main effects of treatment were noted. Total particle concentrations of VLDL and chylomicrons, as well as small VLDL particles, and total LDL and small LDL particle concentrations were significantly lower at the end of the high-dose strawberry phase, compared to values following the low-dose strawberry, baseline, and/or control phases in post hoc analyses (all *p* < 0.0001; Table 4). No significant effects were noted in HDL particle concentrations. On the other hand, HDL particle size was significantly increased after the low-dose strawberry phase, compared to values in the baseline, control, and high-dose strawberry phases in post hoc analyses (*p* < 0.0001; Table 4). No effects were detected with VLDL and LDL particle size. Overall, treatment-by-time interactions were not significant for any of the outcomes except for small LDL particle concentration (*p* = 0.004), indicating that the effect of the treatment on small LDL particles depended on the order in which the treatments were administered.

### 3.4. Adipokines, CRP, and Hormonal Biomarkers

Among the adipokines, a significant treatment effect was observed for serum PAI-1, which revealed significantly lower values following the high-dose strawberry phase, compared to values after the baseline, control, and low-dose strawberry phases in post hoc analyses (*p* = 0.002; Table 5). On the other hand, no effects were observed with serum leptin, resistin, and visfatin (Table 5), as well as for CRP and adiponectin (Table 3). Among the hormonal biomarkers, a significant treatment effect was observed for serum glucagon, which revealed significantly higher values in the high-dose strawberry phase, compared to values in the baseline, control, and low-dose strawberry phases in post hoc analyses (*p* = 0.02; Table 5). No effects were found on serum C-peptide, ghrelin, GIP, and GLP-1 (Table 5). Overall, treatment-by-time interactions were not significant for any of these outcomes except for serum PAI-1 (*p* = 0.003), indicating that the effect of the treatment on PAI-1 depended on the order in which the treatments were administered.

### 3.5. Habitual Dietary Intakes and Physical Activity

No significant differences were noted in the habitual intake of dietary nutrients, as well as total intake of fruits and vegetables for participants throughout the study and across the different phases (Table 6). Similarly, physical activity data showed no significant treatment and time effects in the participants.

## 4. Discussion

The present study demonstrates the role of dietary strawberries at a daily dose of two-and-a-half servings in decreasing serum insulin and concomitant insulin resistance (HOMA-IR), but not fasting glucose in obese adults with features of the metabolic syndrome. Attributable to this decrease in serum insulin, we also observed an increase in serum glucagon and a decrease in serum PAI-1 with this dose of strawberries. While significant improvements were not observed in conventional lipids, the results revealed a decrease in serum LDL cholesterol, which was borderline significant. Further, the higher dose of strawberries also resulted in improvements in lipid particle concentrations, especially in decreasing VLDL and chylomicron particles, as well as total and small LDL particles, all of which have important consequences in decreasing atherogenic risk in these adults. The daily lower dose of dietary strawberries of one serving did not lead to many changes, except an increase in HDL particle size. Given the projected increase in the global incidence of type 2 diabetes [22], in which insulin resistance plays a key role, our findings are of relevance in decisions regarding public health nutrition.

To our knowledge, this is the first study to document the role of strawberries in improving insulin resistance in a nonacute setting for four weeks in at-risk adults. Our findings on the role of high-dose strawberries (~960 mg total polyphenols) in improving insulin resistance are corroborated by several reported clinical studies using different varieties of dietary berries. Using a combination of strawberry and cranberry polyphenols (333 mg for six weeks), Paquette et al. reported significant increases in insulin sensitivity, and a decrease in first-phase insulin secretion when compared to the control group in adults with insulin resistance [23]. In another study, feeding freeze-dried blueberry powder (1462 mg polyphenols for six weeks) also revealed improvements in insulin sensitivity in adults with metabolic syndrome [24]. Other feeding trials using cranberry juice polyphenols (~346 mg polyphenols for eight weeks) have also revealed similar [25,26] but some inconsistent results [27] on insulin resistance and glucoregulation in adults with cardiometabolic risks. In an acute study using a slightly higher dose of strawberries than what was used in our study (40 g strawberry powder; ~three servings of fresh strawberries), Park et al. reported a decrease in six-hour postprandial insulin load when compared to the control group in adults with insulin resistance [28]. Their findings, together with our report, provide evidence that strawberries at a dose of two-and-a-half to three servings a day or when taken with meals could reduce the pancreatic burden of greater insulin secretion by reducing insulin resistance and yet maintaining normal blood glucose levels. These observations have been explained by mechanistic studies reporting the role of strawberry polyphenols and dietary polyphenols for improving insulin signaling and inflammation in animal models of insulin resistance and diabetes [29,30,31,32]. These findings warrant further investigation in adults with type 2 diabetes regarding the use of strawberry polyphenols, alone and in combination with other dietary polyphenols, to examine maximal efficacy on impaired glycemia.

In contrast to our previous findings on the role of freeze-dried strawberry powder in reducing total and LDL cholesterol [15,16], the present study revealed a borderline significant lowering of LDL cholesterol in the high-dose strawberry phase. These differences may be attributed to the shorter duration of each phase in the current study and dose differences when compared to our previous trial. Nonetheless, these improvements within a short duration in adults with elevated LDL cholesterol are of clinical and public health relevance based on the increased global CVD mortality rates that can be attributable to elevated LDL cholesterol [12]. NMR-derived lipid profiles revealed atherogenic profiles that are often not identified in conventional lipids [33], that is, total particle concentrations of VLDL and small LDL particles have been associated with increased CVD risk and have been the target for therapeutic interventions in patient-oriented studies [21,34]. In this context, the present study observations of strawberries decreasing these atherogenic lipid particles confirm our [16] and other [35] previously reported findings. The lipid-lowering effects of strawberries may be explained by the fruit fiber, phytosterols, and a combination of polyphenols that interfere with lipid digestion, absorption, and excretion [36,37]. These confirmatory findings further strengthen the evidence on the role of strawberries in improving CVD risks and preventing disease progression.

Our findings on the effect of high-dose strawberries in decreasing serum PAI-1 levels are novel and are consistent with a single reported acute study in which coadministration of freeze-dried strawberry powder (~95 mg total polyphenols) caused a significant decrease in postprandial PAI-1 levels when compared to those consuming no strawberries [38]. Insulin resistance has been associated with increased expression of PAI-1 by human adipocytes [39] and increased PAI-1 with inflammation and CVD risks [40,41]. Our observed decreases in serum PAI-1 may be attributable to the downstream effects of improved insulin resistance with high-dose strawberry supplementation. Our findings are corroborated by mechanistic studies demonstrating decreased PAI-1 expression by dietary polyphenols [42,43]. On the other hand, we did not observe any differences in other biomarkers of obesity including serum leptin, adiponectin, CRP, resistin, and visfatin, which could be explained by the short duration of each phase of the intervention, and no difference in body weight status in these participants.

Our study has several strengths. Our randomized crossover study design addresses the high interindividual variabilities observed in a parallel trial. Our study simultaneously examined two different dietary achievable doses of whole strawberries administered as freeze-dried powder. This design addressed a gap in dosing consideration since most studies have used higher doses or a single dose of berries that do not reflect habitual consumption and therefore lack sustainability. We report detailed lipid profiles, including concentrations of individual lipid particles and particle size, which have been reported by few clinical studies examining the effects of berry ingestion in adults. Additionally, based on the administration of a control powder that matched the freeze-dried strawberries in sensory qualities, we were able to keep the participants and study coordinators blinded to the test agents. In addition, we excluded participants on medications for lowering lipids and diabetes, as well as those participating in a weight loss program, and thus were able to minimize potential confounding by these factors. In the same context, our study also had a limitation by excluding participants taking medications for lowering blood glucose and lipids, and this may limit the generalizability of our findings to the larger adult population who typically are on these prescriptions. Additionally, although the total study duration was 14 weeks, each phase of the intervention lasted for only four weeks, and this could explain some of the limited effects on cardiometabolic biomarkers. Moreover, we only measured a selected set of inflammatory and adipokine biomarkers, which have been associated with obesity and insulin resistance, and thus, future studies should examine a more comprehensive panel of the types of biomolecules, including antioxidant factors, that would modulate disease outcomes following strawberry supplementation. Finally, the short one-week washout phase between control and treatment phases may have led to some carryover effect, and this limitation is inherent in a crossover study design. Additionally, we did not measure the biomarkers at the end of the washout phase since we do not expect metabolic markers to differ significantly within a week.

In conclusion, our randomized controlled crossover trial supports the hypothesis that dietary strawberries when consumed at a dose of two-and-a-half servings a day significantly improve cardiometabolic risks, mainly via improving insulin resistance and atherogenic lipid particles, when compared to the control group. When consumed at a dose of one serving of strawberries, modest effects were observed for improving HDL particle size. These findings support the role of dietary strawberries in a medical nutrition therapeutic approach for diabetes prevention in adults.

## Figures and Tables

**Table 1:** Composition of test powders consumed per day.

Variable	Control	Strawberry-LD	Strawberry-HD
Weight, g	32	32	32
Calories, Kcal	122	123	124
Total Carbohydrates, g	29	28	27
Ash, g	1.1	0.7	2.0
Dietary fiber, g	3.2	2.1	5.0
Vitamin C, mg	ND	26	65
Total phenolics, mg ^1^	ND	400	960
Total anthocyanins, mg ^2^	ND	38	92
Total ellagic acid, mg	ND	9	25
Total flavan-3-ols, mg	ND	24	60

HD = high dose (~32 g powder or 2.5 servings of strawberries/day); LD = low dose (~13 g powder or 1.0 serving of strawberries/day); ND = not detectable. ^1^ Expressed as mg gallic acid equivalents. ^2^ Expressed as mg cyanidin-3-glucoside equivalents.

**Table 2:** Baseline characteristics of study participants.

N	33
Age, (y)	53 ± 13
Sex, (M/F)	2/31
BMI, (kg/m^2^)	33 ± 3
Blood pressure medication use, n (%)	6 (18)
Antidepressant use, n (%)	8 (24)
Multivitamin use, n (%)	5 (15)
Meeting exercise recommendations (%) ^1^	11 (33)

Data are presented as mean and standard deviations (SD). Count data presented as n (%). M = male; F = female; BMI= body mass index. ^1^ ≥150 min of moderate and/or ≥90 min vigorous exercise/week.

**Table 3:** Cardio–metabolic profiles in obese adults with above optimal serum LDL cholesterol following each treatment period in a randomized crossover study.

Variable	Baseline	Control (4-Week)	Strawberry (LD) (4-Week)	Strawberry (HD) (4-Week)	*p*-Value ^1^ (Treatment)
Body weight, lb	189 ± 23	189 ± 23	190 ± 23	190 ± 23	0.99
BMI, kg/m^2^	32.0 ± 2.5	32.0 ± 2.4	32.0 ± 2.5	32.0 ± 2.4	0.89
Waist circumference, inches	40.0 ± 2.8	41.0 ± 3.0	41.0 ± 3.0	41.0 ± 3.0	0.92
Systolic blood pressure, mm Hg	127 ± 8	126 ± 10	127 ± 9	124 ± 8	0.78
Diastolic blood pressure, mm Hg	82 ± 7	82 ± 8	80 ± 7	80 ± 8	0.84
Serum total cholesterol, mg/dL	221 ± 28	217 ± 28	209 ± 34	208 ± 32	0.24
Serum LDL cholesterol, mg/dL	144 ± 25	139 ± 23	133 ± 29	127 ± 24	0.05
Serum HDL cholesterol, mg/dL	54 ± 10	53 ± 11	53 ± 11	53 ± 9	0.96
Serum LDL:HDL	2.8 ± 0.9	2.8 ± 0.9	2.7 ± 0.9	2.6 ± 0.8	0.77
Serum Triglycerides, mg/dL	124 ± 66	126 ± 60	128 ± 67	133 ± 76	0.95
Serum Fasting glucose, mg/dL	93 ± 13	93 ± 12	94 ± 11	93 ± 15	0.97
Serum HbA1c, %	5.5 ± 0.3	5.5 ± 0.3	5.5 ± 0.3	5.5 ± 0.2	0.95
Serum Insulin, µIU/mL *	15.4 ± 6.6 ^a^	15.2 ± 6.4 ^a^	14.0 ± 8.2 ^a^	9.1 ± 3.1 ^b^	**0.0002**
Serum HOMA-IR	3.6 ± 1.5 ^a^	3.5 ± 1.4 ^a^	3.3 ± 2.0 ^a^	2.1 ± 0.5 ^b^	**0.0003**
Serum hs-CRP, mg/L	4.3 ± 3.2	4.4 ± 3.5	4.3 ± 3.1	3.8 ± 2.9	0.85
Serum adiponectin, µg/mL	9.3 ± 5.7	10.5 ± 6.2	11.4 ± 5.2	11.7 ± 7.2	0.84

Data presented as means ± SD. N = 33/group. BMI= body mass index; HD = high dose (~2.5 servings of strawberries/day); HOMA-IR = homeostasis model assessment of insulin resistance; hs-CRP = high sensitivity C-reactive protein; LD = low dose (~1.0 serving of strawberries/day). ^1^ *p* for main effect of treatment from the MIXED procedure (SAS version 9.4; SAS Institute Inc., Cary, NC, USA) adjusted for baseline values. Different superscript letters show significant differences among treatment groups for each variable. *p* < 0.05 in bold font. * *p* for time effect significant with lower values at 14 weeks, compared to baseline and four weeks (*p* < 0.05).

**Table 4:** Plasma NMR-derived lipid particle concentrations and size in obese adults with above optimal serum LDL cholesterol following each treatment period in a randomized crossover study.

Variable	Baseline	Control (4-Week)	Strawberry (LD) (4-Week)	Strawberry (HD) (4-Week)	*p*-Value ^1^ (Treatment)
VLDL and chylomicron particles (total), nmol/L	39.2 ± 16.3 ^a^	41.7 ± 15.9 ^a^	42.6 ± 16.0 ^a^	32.3 ± 13.0 ^b^	**<0.0001**
Large VLDL and chylomicron particles, nmol/L	6.1 ± 3.9	6.7 ± 3.7	6.8 ± 3.7	6.3 ± 3.2	0.06
Medium VLDL particles, nmol/L	12.9 ± 9.5	14.3 ± 9.3	14.3 ± 9.3	12.7 ± 8.8	0.07
Small VLDL particles, nmol/L	21.8 ± 10.2	22.3 ± 10.8 ^a^	23.1 ± 10.0 ^a^	13.7 ± 5.4 ^b^	**<0.0001**
LDL particles (total), nmol/L *	1126 ± 328	1196 ± 326 ^a^	1204 ± 310 ^a^	1042 ± 297 ^b^	**<0.0001**
IDL particles, nmol/L	341 ± 175	359 ± 173	362 ± 169	357 ± 174	0.12
Large LDL particles, nmol/L	153 ± 99	152 ± 93	152 ± 92	150 ± 93	0.76
Small LDL particles (total), nmol/L *	638 ± 188 ^a^	695 ± 242 ^a,b^	681 ± 219 ^a,b^	535 ± 140 ^b^	**<0.0001**
HDL particles (total), µmol/L	29.1 ± 5.6	29.5 ± 5.8	30.0 ± 5.7	29.5 ± 5.8	0.18
Large HDL particles, µmol/L	4.2 ± 1.9	4.4 ± 1.8	4.5 ± 1.8	4.2 ± 1.7	0.11
Medium HDL particles, µmol/L	5.5 ± 4.7	5.6 ± 4.8	6.0 ± 4.9	5.6 ± 4.7	0.24
Small HDL particles, µmol/L	19.5 ± 5.1	20.1 ± 4.6	20.7 ± 4.1	20.0 ± 4.7	0.22
VLDL size, nm	55.4 ± 7.1	55.5 ± 7.7	55.9 ± 8.0	54.1 ± 7.8	0.35
LDL size, nm	20.1 ± 0.6	20.8 ± 1.8	21.3 ± 1.9	20.2 ± 1.9	0.45
HDL size, nm	9.2 ± 0.5 ^a^	9.2 ± 0.7 ^a^	11.3 ± 2.4 ^b^	9.3 ± 0.7 ^a^	**<0.0001**

Data presented as means ± SD. N = 33/group. HD = high dose (~2.5 servings of strawberries/day); LD = low dose (~1.0 serving of strawberries/day); NMR = nuclear magnetic resonance. ^1^ *p* for main effect of treatment from the MIXED procedure (SAS version 9.4; SAS Institute Inc., Cary, NC, USA) adjusted for baseline values. Different superscript letters show significant differences among treatment groups for each variable. *p* < 0.05 in bold font. * *p* for time effect significant with lower values at 14 weeks, compared to baseline and four weeks (*p* < 0.05).

**Table 5:** Diabetes-related adipokines and hormonal markers in obese adults with above optimal serum LDL cholesterol following each treatment period in a randomized crossover study.

Variable	Baseline	Control (4-Week)	Strawberry (LD) (4-Week)	Strawberry (HD) (4-Week)	*p*-Value ^1^ (Treatment)
Serum C-peptide, pg/mL	1232 ± 929	1273 ± 531	1254 ± 752	1021 ± 680	0.68
Serum ghrelin, pg/mL	486 ± 371	371 ± 361	380 ± 319	482 ± 392	0.62
Serum GIP, pg/mL	478 ± 357	354 ± 274	378 ± 219	564 ± 426	0.23
Serum GLP-1, pg/mL	314 ± 59	265 ± 47	288 ± 69	307 ± 88	0.23
Serum glucagon, pg/mL	654 ± 147	609 ± 93	635 ± 113	725 ± 117	**0.02**
Serum leptin, pg/mL	13736 ± 9204	12795 ± 4841	14884 ± 7433	9547 ± 3635	0.09
Serum PAI-1, pg/Ml *	5621 ± 1022	5812 ± 1240	5245 ± 1278	4315 ± 1434	**0.002**
Serum resistin, pg/mL	10487 ± 6799	11787 ± 6516	11711 ± 6238	10199 ± 4811	0.82
Serum visfatin, pg/mL	3240 ± 1310	2733 ± 1033	2797 ± 954	3176 ± 1352	0.45

Data presented as means ± SD. N = 33/group. GIP = gastric inhibitory polypeptide; GLP-1 = glucagon-like peptide-1; HD = high dose (~2.5 servings of strawberries/day); LD = low dose (~1.0 serving of strawberries/day); PAI-1 = plasminogen activator inhibitor-1. ^1^ *p* for main effect of treatment from the MIXED procedure (SAS version 9.4; SAS Institute Inc., Cary, NC, USA) adjusted for baseline values. Different superscript letters show significant differences among treatment groups for each variable. *p* < 0.05 in bold font. * *p* for time effect significant with lower values at 14 weeks, compared to baseline and four weeks (*p* < 0.05).

**Table 6:** Background daily dietary nutrient and food group intakes in obese adults with above optimal serum LDL cholesterol following each treatment period in a randomized crossover study.

Variable	Baseline	Control (4-Week)	Strawberry (LD) (4-Week)	Strawberry (HD) (4-Week)	*p*-Value ^1^ (Treatment)
Total calories, kcal	2012 ± 152	1988 ± 183	2123 ± 113	2067 ± 193	0.74
Carbohydrates, % kcal	45 ± 6	48 ± 8	44 ± 5	46 ± 8	0.66
Fats, % kcal	34 ± 4	36 ± 7	35 ± 5	37 ± 5	0.45
Proteins, % kcal	20 ± 5	16 ± 6	21 ± 7	17 ± 6	0.42
Total sugars, g *	85 ± 11	78 ± 12	72 ± 9	75 ± 10	0.08
Fiber, g	15 ± 11	18 ± 12	22 ± 15	21 ± 10	0.18
Vitamin E, mg	9 ± 2	11 ± 4	11 ± 2	10 ± 4	0.22
Vitamin C, mg	45 ± 13	46 ± 12	49 ± 12	49 ± 12	0.26
Fruits, cups	1.0 ± 0.4	1.0 ± 0.3	0.8 ± 0.2	1.0 ± 0.4	0.45
Vegetables, cups	1.3 ± 0.2	1.1 ± 0.3	0.9 ± 0.2	1.1 ± 0.4	0.38

Data presented as means ± SD. N = 33/group. HD = high dose (~2.5 servings of strawberries/day); LD = low dose (~1.0 serving of strawberries/day). ^1^ *p* for main effect of treatment from the MIXED procedure (SAS version 9.4; SAS Institute Inc., Cary, NC, USA) adjusted for baseline values. * *p* for time effect significant with lower values at 14 weeks, compared to baseline and four weeks (*p* < 0.05).

## Data Availability

The datasets analyzed in the current study are not publicly available due to ethical reasons and because our participants only gave their consent for the use of their data by the original team of investigators.

## Clinical Trial Registry

Clinicaltrials.gov identifier NCT03441620.
